# Effects of Trace Mineral Source on Growth Performance, Antioxidant Activity, and Meat Quality of Pigs Fed an Oxidized Soy Oil Supplemented Diet

**DOI:** 10.3390/antiox13101227

**Published:** 2024-10-12

**Authors:** Ge Zhang, Jingyi Huang, Zhiqiang Sun, Yuhan Guo, Gang Lin, Zeyu Zhang, Jinbiao Zhao

**Affiliations:** 1 State Key Laboratory of Animal Nutrition and Feeding, College of Animal Science and Technology, China Agricultural University, Beijing 100193, China; zhangge0557@163.com (G.Z.); 13370102152@163.com (J.H.); sunbeibei5421@163.com (Z.S.); mafic2017zeyu@163.com (Z.Z.); 2 Beijing Alltech Biological Products (China) Co., Ltd., Beijing 100600, China; bguo@alltech.com (Y.G.); glin@alltech.com (G.L.)

**Keywords:** oxidized soy oil, organic trace minerals, selenium yeast, meat quality, antioxidant activity, growing–finishing pigs

## Abstract

This study investigates the effects of oil quality and trace mineral source on the growth performance, antioxidant activity, and meat quality of growing–finishing pigs. A total of 180 crossbred pigs (Duroc × Landrace × Large White [64.4 ± 1.95]) were randomly allocated five dietary treatments based on body weight (BW) and sex in a 30 d trial. Pigs were fed five diets: (i) fresh soy oil + inorganic trace minerals (ITMs) + inorganic selenium (FISI), (ii) oxidized soy oil + ITMs + inorganic selenium (OISI), (iii) fresh soy oil + ITMs + selenium yeast (FISY), (iv) oxidized soy oil + ITMs + selenium yeast (OISY), and (v) oxidized soy oil + organic trace minerals (OTMs) + selenium yeast (OOSY). Each dietary treatment included six replicates and six pigs per replicate (three barrows and three gilts). Feeding OISI resulted in lower average daily gain (ADG) and dressing percentage (*p* < 0.05). The OOSY group had a higher dressing percentage and activities of serum CAT and GSH-Px in growing–finishing pigs (*p* < 0.05). In addition, the relative abundance of Campylobacterota in the colonic digesta varied with the quality of soy oil and source of trace minerals (*p* < 0.05), but no significant differences in short-chain fatty acid concentrations were observed among all dietary groups. In conclusion, adding oxidized soy oil to the diet negatively impacted the ADG and dressing percentage of growing–finishing pigs, and replacing ITMs with OTMs and SY alleviated these negative impacts. A combination of OTMs and SY can support antioxidant capacity to mitigate the negative impacts of oxidized oil on the growth performance and dressing percentage of growing–finishing pigs.

## 1. Introduction

Due to the high energy needs of growing–finishing pigs for the deposition of fat and protein, it is fundamental to add an appropriate amount of fats and oils to the diet for their growth and development. In addition to providing energy, fats and oils have other functions in pig production, such as reducing dust in the feed and lessening wear and tear on mixing equipment and feeders [1]. Unfortunately, fats and oils are often oxidized in production settings due to factors such as the storage environment and consideration of feed production cost for farmers. According to previous reports [2], there is a considerable difference in the fatty acid (FA) composition and palatability of oils after oxidation. Moreover, it is well known that the presence of primary oxidation products have negative impacts on feed utilization efficiency, animals’ growth performance, and the quality of animal products [3].

Trace minerals (TMs) are essential nutrients for livestock, and they play a vital role in the various physiological processes of animals. Deficiency in TM results in the poor health and reduced productivity of animals [4]. For instance, manganese (Mn), zinc (Zn), and copper (Cu) are essential micronutrients in oxidative stress regulation pathways. In addition, TMs play critical roles in regulating the functions of endogenous digestive enzymes and hormones in nutrient digestion and absorption. Traditionally, inorganic trace minerals (ITMs) are supplemented to fulfill mineral requirements for optimal growth performance and high-quality animal products. However, ITMs are hygroscopic and reactive, which means they can catalyze oil oxidation and lead to lower bioavailability in the pig’s gastrointestinal tract due to dietary antagonisms. Therefore, it is a common practice in commercial conditions to increase the quantity of the mineral addition, resulting in exceeding the recommendations of the National Research Council (NRC) for modern commercial pigs [5]. However, under normal physiological conditions, only a small amount of dietary iron (Fe), zinc (Zn), manganese (Mn), and copper (Cu) is absorbed, with the remainder being excreted into the environment [6]. Studies on pigs have shown that organic trace minerals (OTMs) have higher bioavailability compared to ITMs, resulting in enhanced mineral absorption, higher mineral concentrations in the circulatory system or tissues [7,8], and less mineral excretion [9,10]. Therefore, adding OTMs to the diet is an effective strategy to reduce the concentrations of trace minerals in the urine and feces of pigs. It has been reported that providing pigs with a mixture of OTMs is superior to individual replacement of ITMs, as there are less interactions in trace mineral absorption among different mineral elements [11]. However, most studies on OTMs for pigs have focused on the influences on pig performance by replacing one or two ITMs [12] and have primarily considered interactions between minerals elements, while interactions between OTMs and oxidized oils have been rarely investigated [10].

Selenium (Se) is a trace element necessary for maintaining animal nutrition and health through its role in biological processes, such as maintaining immune function and regulating oxidation reduction reactions. Se is a major component of 25 types of selenoproteins [13], most of which can improve antioxidant capacity and immune functions [14]. A previous study has shown that there is a significant correlation between the physiological function of Se and amount of Se intake [15]. Insufficient Se intake is associated with low immune function in the host, increased mortality rate, and risk of chronic disease [16]. The metabolic pathways, bioavailability, physiological functions, and toxicity characteristics of Se are related to the amount of Se ingested and the chemical form of this element [17]. Generally, organic Se has higher bioavailability and lower toxicity than inorganic Se [18,19]. Some previous studies indicated that adding organic selenium to daily feed can improve meat quality [20] and increase Se deposition in eggs and broiler tissues [21,22]. Exploring the interactions between the Se source and oil quality can provide insights into solving the problem of dietary oil oxidation.

Therefore, the aim of this study is to investigate the effects of oil quality (fresh and oxidized) and trace mineral source (organic and inorganic) on the growth performance, antioxidant activity, and meat quality of growing–finishing pigs. It also explores whether there is an interaction between trace mineral sources and oxidized oils.

## 2. Materials and Methods

The feeding trial was conducted at the FengNing Swine Research Unit of China Agricultural University (Chengdejiuyun Agricultural and Livestock Co., Ltd., Chengde, China). The study was approved by the Institutional Animal Care and Use Committee of China Agricultural University (CAU AW61404202-1-2, Beijing, China).

### 2.1. Diets and Experimental Design

Based on previous research and the supplier’s recommendations, the growth–finishing pig trace mineral levels for this experiment were as follows: Fe, 80 mg/kg; Cu, 15 mg/kg; Mn, 20 mg/kg; Zn, 80 mg/kg, and Se at 0.3 mg/kg [23,24]. In this study, ITMs in the form of common sulfate salts were used to provide these levels. Inorganic selenium (I-Se) in the form of sodium selenium or selenium yeast (SY; Sel-Plex, Alltech do Brasil Agroindustrial Ltd., Maringá, Brazil) were used to provide 0.3 mg/kg Se. The OTMs were supplied as proteinate (Tianjin Alltech Biological Products Co., Ltd., Tianjin, China) to provide 20 mg/kg Fe, 3.75 mg/kg Cu, 5 mg/kg Mn, and 20 mg/kg Zn in the diet.

The soy oil was purchased from Quanjiafu Co., Ltd. (Langfang, China), and the oxidized oil was prepared by heating [25]. In short, the oil was continuously heated to boiling and bubbling at 100 °C approximately 14 times (each time for 6 h) using an induction cooker. The degree of lipid peroxidation was determined by measuring peroxide value (POV) [26] and acid value [27]. The FA composition of dietary oil was determined using high-performance liquid chromatography (Table 1).

Based on the quality of oil and source of trace minerals, the 5 experimental diets were formulated as follows:

1. Treatment 1: Fresh soy oil + ITMs + I-Se (FISI);
2. Treatment 2: Oxidized soy oil + ITMs + I-Se (OISI);
3. Treatment 3: Fresh soy oil + ITMs + 150 g/t SY (FISY);
4. Treatment 4: Oxidized soy oil + ITMs + 150 g/t SY (OISY);
5. Treatment 5: Oxidized soy oil + 400 g/t OTMs + 100 g/t SY (OOSY).

The information about the sources and amounts of trace minerals are listed in Table 2. The composition of the basal diet is shown in Table S1. Vitamin and mineral premixes without trace elements were added to meet the nutritional requirements of growing–finishing pigs according to the NRC (2012) [28]. The premixes provided for the pigs did not contain trace minerals of Fe, Cu, Mn, Zn, and Se. Dietary minerals were measured using an atomic absorption spectrophotometer according to the method described by Liu et al. [6], and the values are shown in Table 2.

### 2.2. Animals, Housing and Sample Collection

A total of 180 (Duroc × Large White × Landrace) growing–finishing pigs with an initial body weight (BW) of 64.4 ± 1.95 kg were randomly assigned into 5 groups balanced for BW and sex. Each treatment group consisted of 6 replicates with 6 pigs per replicate arranged in 1 pen. Each replicate maintained an equal ratio of males and females. The feeding trial lasted 30 d. Weaned piglets were housed in pens measuring 1.6 m × 1.4 m × 0.8 m, with plastic-slatted floors and equipped with automatic stainless steel nipple drinkers and feeders. All pigs were managed according to standard procedures followed at the pig farm.

On d 1 and d 30 of the experiment, individual BW of pigs and feed intake of each pen were measured, and the average daily gain (ADG), average daily feed intake (ADFI), and feed-to-gain ratio (F: G) were calculated. On d 29, one pig close to the average body weight of pigs in the pen (estimated by visual inspection) was selected and a 5 mL sample of blood was collected via the anterior vena cava using a non-anticoagulant vacuum blood collection tube. The samples were left at room temperature for 2 h and then centrifuged at 3000× *g* for 20 min at 4 °C, and the supernatant was collected and stored at −20 °C for serum parameters analysis. After a 12 h fasting period (d 30), one pig close to the average body weight from each replicate was selected for slaughter to collect tissues at the end of experiment. The *longissimus dorsi* at the left 10th to 11th ribs was taken for pH, meat color and drip loss determination [29]. Liver samples were immediately stored in liquid nitrogen for analysis of antioxidant activity. The remaining liver, abdominal fat, and *longissimus dorsi* muscle samples were collected and stored at −80 °C prior to fatty acids analysis. Fresh colonic digesta samples (5 mL) in each group (*n* = 5) were collected in cryovials and immediately immersed in liquid nitrogen and then stored at −80 °C until analysis of microbial composition and metabolites.

### 2.3. Carcass Traits

After slaughter, carcasses were processed following conventional procedures. Carcass weight was measured after removing head, hooves, tail, and internal organs, while the kidneys and leaf fat were retained. The oblique length of the carcass was measured as the length from the inner edge of the union of the first rib and sternum to the anterior edge of the pubic symphysis. The backfat thickness at the 10th rib on the left half of the carcass was measured using vernier calipers. The length and width of the *longissimus dorsi* was also determined. The dressing percentage and eye muscle area were calculated as follows:

Dressing percentage (%) = carcass weight/the live weight before slaughter;

Eye muscle area (cm^2^) = length × width × 0.7.

### 2.4. Meat Quality Assessment

Meat quality of the *longissimus dorsi*, including shear force, cooking loss, drip loss, pH _45min_, pH _24h_, lightness (L*), redness (a*), and yellowness (b*), was determined after slaughter.

Marbling score and meat color score were evaluated on-site using the marbling score card and the meat color standard score card (National Pork Producer Council, NPPC, Washington, DC, USA). Forty-five minutes after slaughter, the meat color value and pH value of the *longissimus dorsi* muscle were measured using a Colorimeter (50 mm diameter aperture, C illuminant, CR410, Minolta, Tokyo, Japan) and a pH meter (Testo 205, Kirchzarten, Germany) at 4 °C, respectively. A second measurement was taken for the *longissimus dorsi* muscle 24 h after slaughter.

The drip loss was determined according to the method described in a previous study [30]. Briefly, samples of approximately 30 g were cut from the *longissimus dorsi* and then were stored in covered plastic boxes over sieved plastic racks for 48 h at temperatures from 2 to 4 °C. After storage, the samples were re-weighed and the difference in weight was used to determine drip loss. Drip loss percentage was determined as follows:

Drip loss = [(initial weight − 24 h weight)/initial weight] × 100.

### 2.5. Determination of Serum Parameters

The measurement of serum parameters was conducted according to a previously reported method [31], and the activity of total antioxidant capacity (T-AOC) and glutathione peroxidase (GSH-Px) and total superoxide dismutase (T-SOD) and malondialdehyde (MDA) concentration in serum were measured using biochemical methods following manufacturer’s instructions provided for each reagent kit (Nanjing Jiancheng Bioengineering Institute, Nanjing, China). Briefly, the content of MDA was determined by the thiobarbituric acid reaction. The activity of T-AOC, GSH-Px, and T-SOD were assayed using the xanthine oxidase, the dithiodinitrobenzoic acid colorimetric, and ammonium molybdate methods, respectively. Concentrations of interleukin-10 (IL-10), IL-6, and IL-1β in serum were determined using a commercial ELISA kit.

### 2.6. Inflammatory Cytokines and Antioxidant Capacity in the Liver

The levels of antioxidant parameters including SOD, T-AOC, CAT, and GSH-Px were determined using the assay kits (Nanjing Jiancheng Bioengineering Institute, Nanjing, China) following the manufacturer’s instructions for testing.

### 2.7. Short-Chain Fatty Acids Concentrations in Colonic Digesta

The short-chain fatty acid (SCFA) concentrations in colonic digesta were determined following previously reported methods, with appropriate modifications [32]. Briefly, digesta samples were homogenized and diluted with distilled water. After mechanical shaking and centrifuging at 5000× *g* for 15 min, a mixture of supernatant fluid and 25% meta-phosphoric acid solution (3.6 mL:0.4 mL) was allowed to stand overnight. Afterwards, the solution was centrifuged at 5000× *g* for 15 min and filtered through a membrane filter (pore size 0.45 μm). The SCFA concentrations of these samples were determined with gas chromatography (Agilent Technologies 7890B GC System; Agilent, Santa Clara, CA, USA) and DB-FFAP column (30 m × 250 μm × 0.25 μm). Nitrogen was the carrier gas with a flow rate of 0.8 mL/min. A flame ionization detector was used to determine acetate, propionate, butyrate, isobutyrate, valerate, and isovalerate concentrations.

### 2.8. Fatty Acids in the Longissimus Dorsi and Abdominal Fat

The *longissimus dorsi* was cut into 2 mm slices and placed into aluminum boxes, and then was freeze-dried (Tofflon Freezing Drying Systems, Shanghai, China). Lyophilized meat was subsequently ground into powder to analyze the intramuscular fat concentration by using Soxhlet petroleum ether extraction (XT15 Extractor, Ankom Technology Corp., Macedon, NY, USA) as described by Zhang et al. [33]. Concentrations of FA were determined using classical gas chromatography (6890 Series, Agilent Technologies, Wilmington, DE, USA) as described in a previous report [34].

### 2.9. Colonic Microbiota Composition

According to the method described by Wang et al. [35], a total bacterial genomic DNA was extracted from fecal samples using a kit (MP Biomedicals, Santa Ana, CA, USA). The extracted DNA was quantified using NanoDrop 2000 (Thermo Fisher Scientific, Waltham, MA, USA). The quality of the DNA was evaluated using agarose gel electrophoresis. Using the ABI GeneAmp 9700 PCR Thermal Cycler (ABI, Los Angeles, CA, USA), the V3-V4 region of the bacterial 16S rRNA gene was amplified using the universal primers 338F (5′-ACTCCTACGGGAGGCAGCAG-3′) and 806R (5′-GACTACHVGGGTWTCTAAT-3′). Amplicons libraries were purified, quantified, pooled, and sequenced on the Illumina MiSeq PE300 platform (Illumina, San Diego, CA, USA) according to the standard protocol of Majorbio Bio-Pharm Technology Co., Ltd. (Shanghai, China).

### 2.10. Statistical Analysis

Data of growth performance, serum marker concentrations, meat quality, and FA concentrations were checked for normality and outliers using the UNIVARIATE procedure of SAS 9.4 (SAS Institute Inc., Cary, NC, USA). These data were then analyzed using the GLM procedure, and Tukey ‘s test was used to adjust the multiple comparisons; means were separated using the LSMEANS statement. Pens served as the experimental unit for growth performance and nutrient digestibility data. Individual pigs were considered to be the experimental unit for the other indexes. The fixed effect was the dietary treatments, and pig body weight and sex were considered as random effects. The microbiota sequencing data were analyzed on the online platform of Majorbio Cloud Platform (www.majorbio.com). For the microbiota sequencing data, the raw Illumina fastq files underwent demultiplexing and quality filtering using QIIME 2.0 [36]. Alpha diversity was evaluated using the Chao1 index, while beta diversity was visualized using principal coordinates analysis (PCoA). The linear discriminant analysis (LDA) effect size (LEfSe) algorithm was utilized to identify taxa with significantly different relative abundances. The threshold for the logarithmic LDA score was set at 3.0. The Kruskal–Wallis method was employed to analyze the relative abundance of gut microbiota composition. For all analyses, differences were considered significant at *p* < 0.05, and values of 0.05 ≤ *p* < 0.10 were defined as a trend.

## 3. Results

### 3.1. Characterization of the Oils

The oxidation characteristics and fatty acid composition of soy oil are shown in Table 1. The oxidized oil, produced by heating and boiling, had a higher peroxide value (POV) (3.89 vs. 83.58 mmol/kg) and acid value (0.18 vs. 1.17 g KOH/kg). The oxidation of soy oil reduced the concentration of PUFA and increased concentrations of SFA and MUFA.

### 3.2. Growth Performance

**Figure 1:**
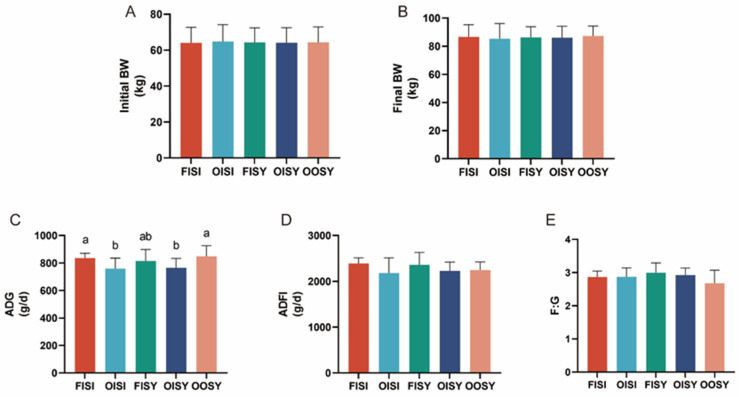
Effects of the quality of oil and source of trace minerals on growth performance in growing–finishing pigs: (**A**) initial body weight (BW); (**B**) final body weight (BW); (**C**) average daily gain (ADG); (**D**) average daily feed intake (ADFI); (**E**) gain-to-feed ratio (G: F). The values are expressed as the means ± SD; bars with different letters are different from each other, *p* < 0.05. FISI: fresh soy oil + ITMs + I-Se; OISI: oxidized soy oil + ITMs + I-Se; FISY: fresh soy oil + ITMs + SY; OISY: oxidized soy oil + ITMs + SY; OOSY: oxidized soy oil + OTMs + SY.

There was no difference in final weight among treatments (Figure 1B). There were also no significant differences in the ADFI or F: G of the pigs among different dietary groups (Figure 1D,E, *p* > 0.05). However, the use of oxidized oil and ITMs together resulted in lower ADG, irrespective of Se source (Figure 1C) (*p* = 0.05).

### 3.3. Carcass Characteristics

The effects of soy oil quality and trace mineral source on slaughter performance are shown in Table 3. In the experiment, there was a significant difference (*p* < 0.01) in the dressing percentage, with the lowest yield of 64.37% observed when using oxidized oil and ITMs. Adding only SY did not significantly alter the slaughter yield (FISY vs. FISI, OISY vs. OISI), but we noted that the inclusion of OTMs and SY mitigated this effect (OOSY vs. OISI, *p* < 0.05). In ITM-fed pigs, the thickness of the sixth to seventh rib fat tended to be greater with oxidized oil compared to fresh oil (*p* = 0.08). No other carcass measures were significantly different among treatments (*p* > 0.05).

### 3.4. Meat Quality

As shown in Table 4, there were no significant differences for meat quality between the ITM- and OTM-fed pigs (*p* > 0.05). By comparing FISI and FISY, we observed that the addition of SY to the diets of growing to finishing pigs, which contained fresh oils and inorganic trace elements, increased a* _45min_ (*p* < 0.05) of the *longissimus dorsi*.

### 3.5. Fatty Acids

The concentration of short-chain fatty acids in the colonic digesta showed no significant differences among treatments (Supplementary Table S2, *p* > 0.05). Regarding the FA composition of the *longissimus dorsi* (Table 5), no significant differences were found among dietary treatments (*p* > 0.05). The FA composition of abdominal fat (Table 6) showed significantly lower content of C22:0 (*p* < 0.05) with utilization of oxidized oil (OISI vs. FISI). Within the oxidized oil treatments, the addition of SY or SY in combination with OTMs resulted in significantly higher C22:0 content in the abdominal fat (*p* < 0.05).

### 3.6. Serum Parameters

**Figure 2:**
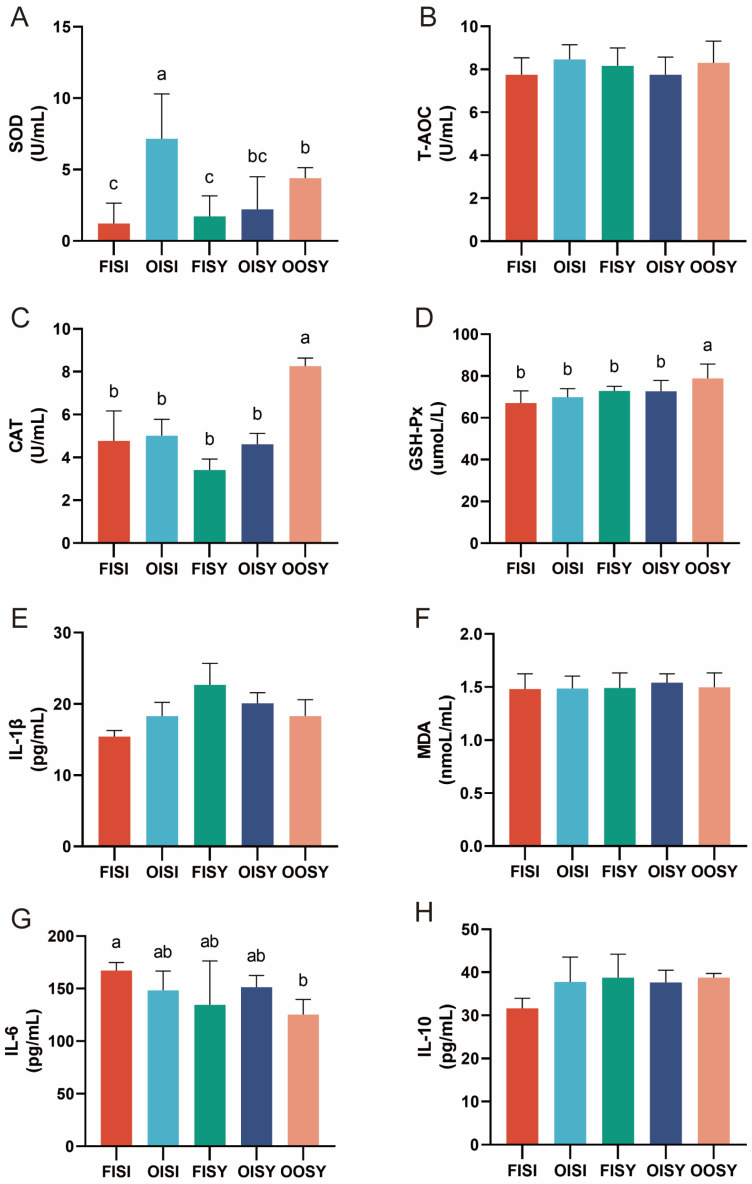
Effects of the quality of oil and source of trace minerals on antioxidase activity and inflammatory cytokines in the serum of growing–finishing pigs: (**A**) SOD; (**B**) T-AOC; (**C**) CAT; (**D**) GSH-Px; (**E**) MDA; (**F**) IL-1β; (**G**) IL-6; (**H**) IL-10. The values are expressed as the means ± SD; bars with different letters are different from each other, *p* < 0.05. FISI: fresh soy oil + ITMs + I-Se; OISI: oxidized soy oil + ITMs + I-Se; FISY: fresh soy oil + ITMs + SY; OISY: oxidized soy oil + ITMs + SY; OOSY: oxidized soy oil + OTMs + SY.

As shown in Figure 2, there was a significant difference in the activity of SOD (Figure 2A) in the serum between fresh and oxidized soy oil when the ITM+I-Se was used in the diet (FISI vs. OISI, *p* < 0.05). The use of OTMs resulted in significantly higher concentration of CAT (Figure 2C) in the serum of the oxidized soy oil group (OISY vs. OOSI, *p* < 0.05), but CAT and GSH-Px were not different among the groups with different oil quality. It is worth noting that the use of OTMs and SY increased the activities of CAT in the serum and T-AOC in the liver of growing and fattening pigs (OOSY vs. OISY, *p* < 0.05). Oxidized oil had no effect on serum IL6 (OISY vs. FISI), but the addition of OTMs and SY significantly reduced serum IL6 levels when fed with oxidized oil (Figure 2G) (*p* < 0.05). In addition, there were no significant differences (*p* > 0.05) observed for serum concentrations of T-AOC, MDA, IL-1β, and IL-10 among the treatments (Figure 2B,E,F,H, respectively).

### 3.7. Liver Antioxidant Activity

**Figure 3:**
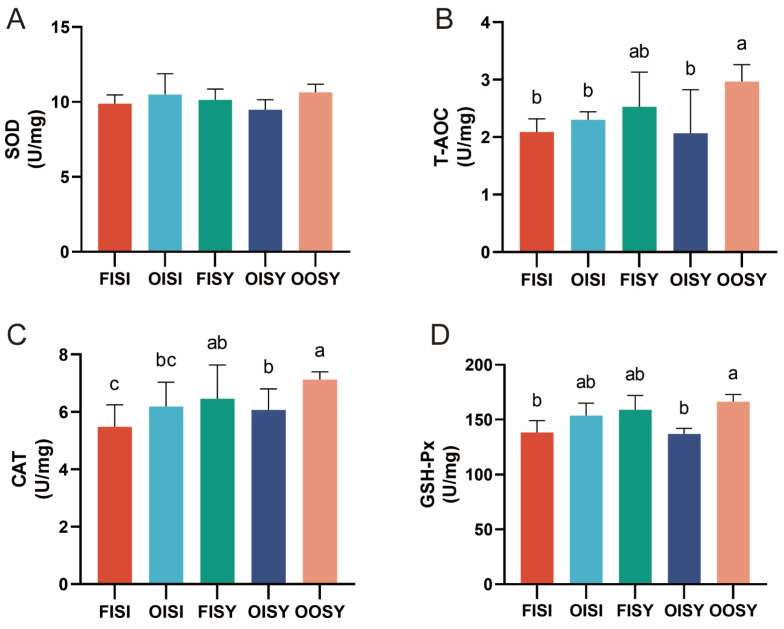
Effects of the quality of oil and source of trace minerals on antioxidant activity in the liver of growing–finishing pigs: (**A**) SOD; (**B**) T-AOC; (**C**) CAT; and (**D**) GSH-Px. The values are expressed as means ± SD; bars with different letters are different from each other, *p* < 0.05. FISI: fresh soy oil + ITMs + I-Se; OISI: oxidized soy oil + ITMs + I-Se; FISY: fresh soy oil + ITMs + SY; OISY: oxidized soy oil + ITMs + SY; OOSY: oxidized soy oil + OTMs + SY.

The results of the impact of oil quality and trace mineral sources on antioxidant enzyme activity in the liver of growing–finishing pigs are shown in Figure 3. In the liver, the concentration of SOD did not differ among dietary treatments (Figure 3A, *p* > 0.05). Consistent with the results of serum antioxidant activity, the activities of T-AOC, CAT, and GSH-Px were higher in the liver of growing–finishing pigs when OTMs were combined with SY in oxidized oil (Figure 3B–D) (OISY vs. OOSI, *p* < 0.05).

### 3.8. Gut Microbial Composition

**Figure 4:**
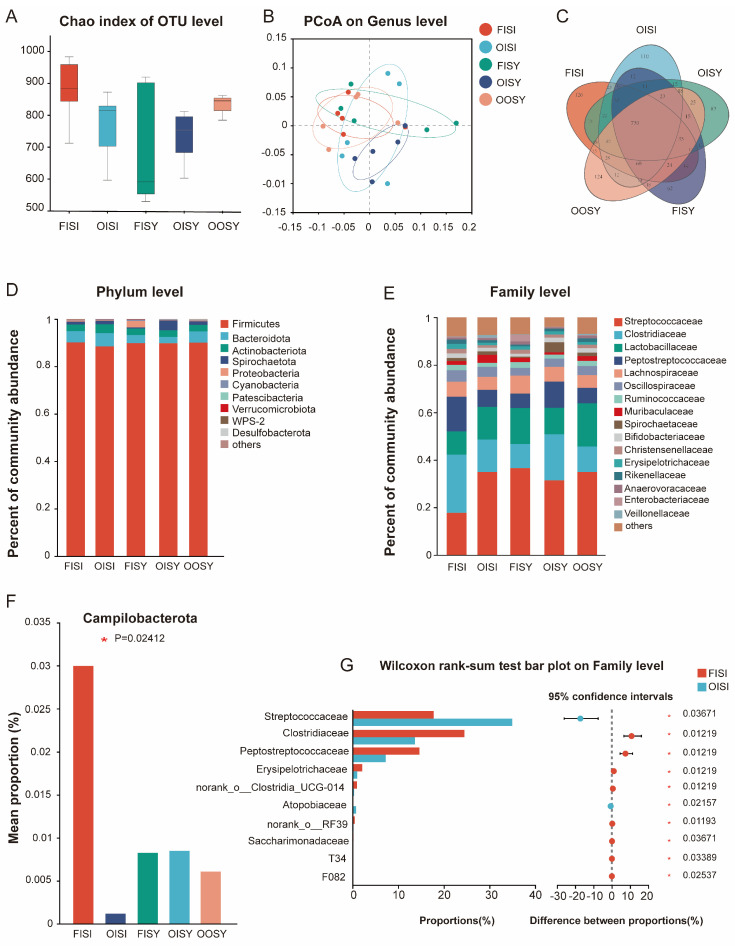
Effects of the quality of oil and the source of trace minerals on gut microbial composition and structure in growing–finishing pigs: (**A**) Chao index; (**B**) PCOA on genus level; (**C**) Venn plot; (**D**,**E**) community bar plot analysis at the phylum level and family levels among all dietary groups; (**F**) the differential bacterial at the phylum level among all dietary groups; (**G**) the differential bacterial at the family level among the FISI and OISI groups. FISI: fresh soy oil + ITMs + I-Se; OISI: oxidized soy oil + ITMs + I-Se; FISY: fresh soy oil + ITMs + SY; OISY: oxidized soy oil + ITMs + SY; OOSY: oxidized soy oil + OTMs + SY.

A microbial α-diversity analysis of the colonic digesta showed no significant differences in the Chao1 index among all treatment groups (Figure 4A). Similarly, PCoA revealed no significant clustering characteristics in the composition of gut bacteria among the treatment groups (Figure 4B). The Venn plot (Figure 4C) shows that the FISI group has the most unique species, with a total of 126 OTUs, followed by 124 and 110 for the OOSY and OISI groups, respectively, and 83 and 62 for the FISY and OISY groups, respectively. The bacterial compositions of fecal microbes at the phylum and family levels are shown in Figure 4D,E, respectively. Results show that the relative abundance of Campylobacterota was affected by the quality of oil and source of trace minerals (Figure 4F). Moreover, compared with the FISI group, the abundances of Streptococcaceae and Atopobiaceae in the OISI group were significantly higher (Figure 4G, *p* < 0.05), while the abundances of Clostridiaceae, Peptostreptococcaceae, and Erysipelotrichaceae were significantly lower (*p* < 0.05, Figure 4G).

## 4. Discussion

Due to the energy requirements of growing and fattening pigs, their daily ration formulas usually require the addition of oil, which may become oxidized after long-term storage. Boler et al. [37] reported that oxidized corn oil significantly reduced ADG and ADFI in finishing pigs, and the addition of synthetic antioxidants (a blend of tert-butylhydroquinone and ethoxyquin) in the diet could not recover the performance decline caused by oxidized soy oil. In the present study, compared with diets containing fresh soy oil, diets supplemented with oxidized soy oil significantly reduced pig ADG. Some studies have focused on the digestibility of nutrients to explain the reduced growth performance when animals were fed the diet containing oxidized oil [38,39]. In those studies of growing and finishing pigs, the addition of oxidized oils led to a decrease in the digestibility of GE and EE, and it is noteworthy that a reduction in ADG was observed in this experiment, but there were no differences in ADFI. Previous studies have shown that the inclusion of OTMs in the diet can enhance the intestinal epithelial barrier function, thereby facilitating mineral metabolism [40,41]. The morphology of the gut not only reveals insights into gut health, but also reflects the collective digestive and absorption capabilities. For instance, an increase in the height of intestinal villi indicates a larger absorptive surface area, suggesting enhanced digestion and absorption of nutrients [42]. Therefore, the increment of ADG in the present study suggested that the addition of OTMs and SY in the diet containing the oxidized soy oil could support pig weight gain by aiding nutrient digestibility and mineral metabolism. A meta-analysis by Hung et al. [43] showed that out of 65 studies on oxidized oils, 48 studies found no significant effect on the pig ADFI, which is consistent with the results of our findings. In another study, Hanson et al. [44] found that the treatment group fed with oxidized lipid diets had lower ADG, ADFI, and G: F by 11.4%, 8.8%, and 3.4%, respectively, compared with the group fed a diet with non-oxidized lipids. It is speculated that this could be due to lower palatability caused by aldehyde compounds generated during the process of lipid oxidation [37,45]. The presence of aldehydes could also be related to the oxidation of vegetable oils (such as soy oil and corn oil) in the diet, which has been associated with decreased growth performance and meat quality in growing–finishing pigs [38,39], although neither of these studies found a significant decrease in ADFI.

Considering production costs, the form of the ITM is usually considered an inexpensive dietary supplement. However, recent studies have shown that when using OTMs instead of ITMs, the return on investment is much higher [46]. There are significant differences in the degree to which OTMs and ITMs are absorbed and utilized by animals, and these differences can ultimately be reflected in the growth performance of the animals. Leeson et al. [47] showed that feeding OTMs at 20% of the ITM level did not affect growth performance. In our study, the ADG of the OOSY group was significantly higher than that of the OISY group and equal to the FISI group. This suggests that the high bioavailability of OTMs remains effective even when added to feed containing oxidized oils. OTMs are more effectively absorbed through amino acid or peptide transport pathways than ITMs, which are absorbed by general mineral absorption pathways [48], which could be the reason for an increment of pig ADG in the OOSY group. Conversely, recent research has shown that the growth performance of piglets was not affected by replacing dietary ITMs with different levels of OTMs [49]. In this study, the utilization of oxidized oils in the feed may have led to the lower feed palatability. Conversely, metabolomics research reveals that oxidized oils, by stimulating the tryptophan–NAD+ metabolic pathway, directly or indirectly diminish the levels of antioxidants (glutathione and ascorbic acid) within the body [50]. This reduction in antioxidant levels, in turn, negatively impacts growth performance, findings that align with this study’s observations. Notably, this study did not observe an increase in oxidative stress induced by the oxidized oils, a topic that will be explored in subsequent sections. Overall, the addition of oxidized soy oil in the diet had a negative impact on the ADG of growing–finishing pigs, which may be attributed to the reduction in the body’s antioxidant levels induced by the oxidized soybean oil.

The addition of oxidized soy oil in the diet has been found to alter carcass characteristics and lower dressing percentage in previous reports [51]. This study also shows that feeding oxidized oil with ITMs results in the lowest value of dressing percentage, which was significantly increased by the addition of OTMs and SY. The composition of dietary fatty acids plays an important role in affecting quality of pork [52,53]. Enhancing meat quality of pigs by adding fatty acids to the diet is a consumer-accepted and effective strategy. However, our results show that the addition of oxidized oil in the diet did not have a significant effect on meat quality (FISI vs. OISI). Another study suggests that adding oxidized corn oil to the diet lowers the dressing percentage and the thickness of the fat on the tenth rib [37]. Dressing percentage was also lower in the present study, and we speculate that oxidized soy oil may increase visceral fat, leading to a lower dressing percentage. Anjum et al. [54] indicated that the liver weight following treatment with oxidized oil was 8% higher compared to that with fresh soybean oil. The visceral organs, being components that are typically discarded during the slaughtering process, can thus influence the overall slaughter yield.

The results of this study show that when the ITMs in the oxidized soy oil group were replaced by OTMs (OISY vs. OOSI), the quality of meat was not significantly changed. However, another study reveals that reducing amounts of inorganic elements (Cu decreased from 3.0 mg/kg to 2.4 mg/kg, Fe from 39.0 mg/kg to 31.2 mg/kg, Mn from 2.1 mg/kg to 1.7 mg/kg, and Zn from 40.0 mg/kg to 32.0 mg/kg) in the diet can significantly impair the quality of pork [55]. The deficiency of trace elements could lead to abnormal physiological responses, such as decreasing the absorption of amino acids and fatty acids, thereby affecting the quality of meat. Zhang et al. [20] indicated that adding organic selenium to the feed increased back fat thickness, skin thickness, and the proportion of belly fat ratio. In our present study, a* of the *longissimus dorsi* muscle of growing–finishing pigs 45 min following slaughter was higher when SY was added to diets with fresh soy oil, which implies that the content of myoglobin was higher in the muscle [56]. Similar results were obtained in a feeding trial regarding compound mineral elements where supplementing compound mineral elements in the diet affected the fatty acid composition of meat in fattening pigs, thereby affecting the quality of the meat [57]. Overall, when fresh oil is administered, SY has an impact on meat color, whereas feeding oxidized oil does not result in differences in ITMs and OTMs. The effect of TMs on meat quality has varied in previous reports, which could be related to the type of trace elements (Se or Cu) and their sources (organic or inorganic).

A previous study has shown that an improvement in antioxidant capacity led to an increase in the a* value of carcass characteristics by reducing the oxidation of pork after slaughter [58]. The above observation matches with observations in the present study, where diets with SY had higher a* _45min_ in the *longissimus dorsi* and enhancement of antioxidant capacity values. In the present study, the use of oxidized soy oil (FISI vs. OISI) resulted in a significant increase in serum SOD activity. The antioxidase activity, such as SOD, CAT, and T-AOC, are important parameters for evaluating antioxidant capacity, while the capacity of non-enzymatic antioxidant defense systems is typically measured using the T-AOC [31,59]. However, a previous study reported that the addition of oxidized corn oil to the diet for laying hens increased oxidative stress and decreased antioxidant enzyme activity in the chicken ovaries [25]. Gowanlock et al. [24] reported that adding OTMs to the diet resulted in higher SOD activity in pig liver and indicated that SOD works in synergy with the selenium-dependent enzyme GSH-Px as part of the antioxidant system. Zhang et al. [20] suggested that organic selenium provides better improvements in meat quality than inorganic selenium, a perspective that aligns with the outcomes of our study. In comparison to the ITM group, the use of OTMs resulted in significantly higher GSH-Px, T-AOC, and CAT in the oxidized oil group (OISY vs. OOSY). Some studies have found that feeding oxidized oil can stimulate and enhance the activity of antioxidant enzymes in the liver [60,61], while other work has reported a decline in antioxidant enzymes [62]. Alterations in hepatic antioxidant enzyme activity might represent a compensatory response, whereby organisms rapidly augment their antioxidant capabilities in response to oxidative stress, aiming to counteract the decline in oxidative damage, such as that inflicted by oxidized lipids [63]. In these experiments, the focus on distinct types of oxidized oils as primary variables was complemented by the observation of variations in the experimental animals and the length of treatment. This leads us to conclude that the effect of different oxidized oils on the antioxidant capacity of various organisms could potentially depend on the duration of the treatment. Notably, the inclusion of OTMs and SY in both experimental setups has significantly contributed to enhancing the organisms’ antioxidant capacities.

Fat is an essential dietary nutrient that is primarily digested and absorbed in the duodenum and jejunum of the small intestine, rather than being digested and absorbed in the large intestine [64]. Although the absorption of dietary fats is nearly entirely confined to the small intestine, upon substantial consumption, some fats do reach the colon, where these minute quantities can still exert an influence on colonic microbial ecology [65]. Different saturations of dietary oils have a significant impact on the composition of the gut microbiota in mammals [66]. Some studies suggest that gavage with oxidized rapeseed oil results in a notable diminution of microbial community diversity within rats [67]. The current experimental results show no difference in α-diversity of gut microbiota among treatment groups, which seems to be related to the type of oil used. Fatty acids and triglycerides, liberated through the hydrolysis of fats, are harnessed by lipolytic bacteria to produce short-chain fatty acids, which serve as an energy source for both bacteria and enterocytes [68], while medium-chain fatty acids can alter the permeability of susceptible strains and thus have antibacterial activity [69]. Fresh and oxidized soy oil have different fatty acid compositions, likely causing different responses in microbial composition. In our study, differences in microbial composition across different oil treatments could be caused by differences in the quantity of hydrolysis products. Notably, Wang et al. [50] discovered, during their metagenomic analysis, that oxidized oils reduced levels of certain metabolites associated with the microbial metabolism. This suggests that oxidized fats not only affect antioxidant processes through the tryptophan–NAD+ pathway, but may also impact microbial ecology. In addition to oil, the varying utilization rates of minerals by bacteria may also have different effects on the gut microbiota of animals [70]. The experiment found the highest abundance of phylum Campylobacterota with OTMs. The metabolites related to Campylobacterota are primarily enriched in d-glutamate and the d-glutamine metabolism, which is always considered to be one of the pathogens and impacts the structure and function of the bacterial cell wall and plays a role in nitrogen metabolism, thereby affecting the host’s intestinal health [71]. In the present study, dietary oxidized oil use resulted in a higher abundance of Streptococcaceae in the gut microbiota, which is associated with metabolic disorders and the occurrence of colon cancer [72]. The impacts of fresh and oxidized soy oil combined with ITMs or OTMs on the composition of the gut microbiota could be associated with the oil digestibility and composition.

## 5. Conclusions

The inclusion of oxidized soy oil in the diet resulted in lower ADG and dressing percentage in growing–finishing pigs, but the negative effects above were at least partially alleviated by the addition of OTMs and/or SY. In addition, dietary supplementation of oxidized soy oil was related to higher levels of saturated fatty acids and lower PUFAs in muscle tissue, resulting in lower meat quality. Inclusion of OTMs positively benefited these measurements of the dressing percentage and also supported antioxidant capacity. Therefore, providing OTMs and SY in the feed when oxidized soy oil is used in pig production may be a reasonable strategy to optimize performance.

## Figures and Tables

**Table 1:** Characteristics of fresh and oxidized soy oils.

Items	Fresh Soy Oil	Oxidized Soy Oil
POV, mmol/kg	3.89	83.58
Acid value, mg KOH/g	0.18	1.17
Fatty acids composition, mg/kg		
SFA	161.37	252.15
C8:0	0.05	0.71
C10:0	0.11	0.31
C12:0	0.14	0.50
C13:0	0.03	0.00
C14:0	0.82	4.83
C15:0	0.11	0.52
C16:0	106.23	172.60
C17:0	0.10	1.66
C18:0	41.32	60.29
C20:0	4.23	3.28
C21:0	0.83	2.01
C22:0	4.83	3.49
C23:0	0.84	0.62
C24:0	1.73	1.33
MUFA	239.34	320.79
C14:1	0.03	0.35
C16:1	0.92	12.26
C18:1n9c	235.72	302.98
C20:1	2.42	4.12
C22:1n9	0.22	0.70
C24:1	0.03	0.38
PUFA	598.86	388.21
C18:2n6c	527.21	347.64
C18:3n3	71.32	38.20
C20:3n6	0.03	0.49
C20:4n6	0.02	0.94
C20:3n3	0.04	0.29
C20:5n3	0.06	0.29
C22:2	0.11	0.35
C22:6n3	0.07	0.00
SUM	999.57	961.14

SFA (saturated fatty acids): C8:0 + C10:0 + C12:0 + C13:0 + C14:0 + C15:0 + C16:0 + C17:0 + C18:0 + C20:0 + C21:0 + C22:0 + C23:0 + C24:0. MUFA (monounsaturated fatty acids): C14:1 + C16:1 + C18:1n9c + C20:1 + C22:1n9 + C24:1. PUFA (monounsaturated fatty acids): C18:2n6c + C18:3n3 + C20:3n6 + C20:4n6 + C20:3n3 + C20:5n3 + C22:2 + C22:6n3.

**Table 2:** The sources and amounts of trace minerals and the quality of oils in the diet ^1^.

Items	FISI	OISI	FISY	OISY	OOSY
Trace minerals, mg/kg					
Fe	80(I)	80(I)	80(I)	80(I)	20(O)
Cu	15(I)	15(I)	15(I)	15(I)	3.75(O)
Mn	20(I)	20(I)	20(I)	20(I)	5(O)
Zn	80(I)	80(I)	80(I)	80(I)	20(O)
Se	0.3(I)	0.3(I)	0.3(O)	0.3(O)	0.3(O)
Dietary inclusion of soy oil, %					
Fresh soy oil	2	-	2	-	-
Oxidized soy oil	-	2	-	2	2
Analysis of dietary minerals, mg/kg					
Fe	82	83	83	82	23
Cu	16	17	17	17	4
Mn	20	20	21	20	6
Zn	78	82	81	80	22
Se	0.30	0.32	0.32	0.31	0.30

^1^ I, trace elements in inorganic form; O, trace elements in organic form.

**Table 3:** Effects of oil quality and of trace mineral source on slaughter performance in growing–finishing pigs.

Items	FISI	OISI	FISY	OISY	OOSY	SEM	*p*-Value
Carcass weight, kg	78.34	77.93	76.31	78.12	79	1.63	0.35
Carcass length, cm	83.55	83.14	81.52	82.78	84.33	1.85	0.14
Dressing percentage, %	66.01 ^bc^	64.37 ^c^	68.40 ^ab^	66.65 ^bc^	69.95 ^a^	1.25	0.01
Loin eye length, cm	101.06	95.49	97.41	99.73	101.87	6.31	0.4
Loin eye width, cm	71.74	65.78	68.84	70.11	71.55	5.73	0.39
Loin eye area, cm^2^	5081	4406	4726	4918	5124	634	0.3
Shoulder fat thickness, mm	28.25	31.96	28.91	30.9	30.82	3.34	0.31
The last rib fat thickness, mm	15.93	16.17	15.36	16.81	17.39	2.99	0.8
Lumbosacral fat thickness, mm	10.25	12.59	11.11	11.07	12.14	2.39	0.47
The 6th to 7th rib fat thickness, mm	19.88	23.37	21.25	24.32	25.02	3.47	0.08
The 10th rib fat thickness, mm	16.04	18.15	16.83	17.86	18.28	3.64	0.79

^a–c^ Different superscript within a row means significant different (*p* < 0.05); FISI: fresh soy oil + ITMs + I-Se; OISI: oxidized soy oil + ITMs + I-Se; FISY: fresh soy oil + ITMs + SY; OISY: oxidized soy oil + ITMs + SY; OOSY: oxidized soy oil + OTMs + SY.

**Table 4:** Effects of the quality of oil and source of trace minerals on meat quality in growing–finishing pigs.

Items	FISI	OISI	FISY	OISY	OOSY	SEM	*p*-Value
Marbling score	2.92	2.25	2.42	3.08	2.75	0.75	0.32
Flesh color score	3.17	3.08	3.17	2.83	3.08	0.67	0.91
l* _45min_	46.82	46.8	47.21	47.02	47.95	2.99	0.96
a* _45min_	17.86 ^b^	18.11 ^b^	18.97 ^a^	18.26 ^ab^	17.65 ^b^	0.7	0.03
b* _45min_	1.01	1.08	0.88	0.92	1.2	0.42	0.7
pH _45min_	5.98	5.93	6.02	6.03	5.89	0.28	0.9
l* _24h_	50.95	49.84	51.92	51.14	52.63	1.76	0.11
a* _24h_	17.63	17.96	17.93	17.64	16.91	0.71	0.09
b* _24h_	0.91	0.98	0.77	0.81	1.12	0.46	0.7
pH _24h_	5.74	5.73	5.74	5.78	5.72	0.17	0.98
Drip loss%	2.42	2.57	2.4	2.58	2.4	0.74	0.98

^a,b^ Different superscript within a row means significant different (*p* < 0.05); FISI: fresh soy oil + ITMs + I-Se; OISI: oxidized soy oil + ITMs + I-Se; FISY: fresh soy oil + ITMs + SY; OISY: oxidized soy oil + ITMs + SY; OOSY: oxidized soy oil + OTMs + SY.

**Table 5:** Effects of the quality of oil and source of trace minerals on FA composition in the *longissimus dorsi* muscle of growing–finishing pigs.

Items, mg/kg	FISI	OISI	FISY	OISY	OOSY	SEM	*p*-Value
SFA	27.72	28.6	26.84	27.45	23.85	1.63	0.93
C8:0	0.02	0.02	0.01	0.02	0.02	0	0.60
C10:0	0.04	0.06	0.04	0.06	0.01	0.01	0.73
C12:0	0.06	0.07	0.06	0.06	0.05	0	0.69
C14:0	0.21	0.1	0.3	0.13	0.32	0.06	0.87
C15:0	3.41	2.68	4.96	2.52	6.54	0	0.84
C16:0	0.01	0.01	0.02	0	0.01	1.04	0.90
C17:0	0.21	0.21	0.22	0.22	0.21	0.01	0.99
C18:0	9.22	9.09	8.99	8.93	8.01	0.5	0.96
C20:0	0.02	0.03	0.09	0.04	0.09	0.01	0.98
C21:0	0.05	0.04	0.12	0.06	0.09	0.02	0.52
C22:0	0.05	0.06	0.06	0.06	0.06	0	0.53
C23:0	0.04	0.04	0.03	0.04	0.04	0	0.61
C24:0	0.05	0.05	0.05	0.06	0.06	0	0.88
MUFA	28.73	32.06	27.53	30.22	25.04	2.11	0.90
C14:1	0.01	0.02	0.01	0.02	0.02	0	0.46
C16:1	1.89	2.38	1.76	2.07	1.68	0.16	0.70
C18:1n9c	26.19	28.96	25.14	27.45	22.73	1.91	0.91
C20:1	0.48	0.55	0.46	0.52	0.43	0.44	0.93
C22:1n9	0.1	0.09	0.1	0.12	0.12	0.01	0.50
C24:1	0.05	0.07	0.06	0.06	0.07	0	0.31
PUFA	19.01	21.17	23.67	19.52	18.07	0.92	0.33
C18:2n6c	8.18	9.04	10.18	8.37	7.55	0.42	0.32
C18:3n3	0.21	0.3	0.41	0.29	0.24	0.03	0.29
C20:3n6	0.2	0.21	0.2	0.21	0.23	0.01	0.60
C20:4n6	1.53	1.69	1.75	1.5	1.73	0.04	0.26
C20:3n3	0.06	0.07	0.08	0.08	0.06	0.01	0.76
C20:5n3	0.06	0.06	0.06	0.06	0.07	0	0.84
C22:2	0.05	0.07	0.05	0.05	0.05	0.07	0.87
C22:6n3	0.08	0.09	0.09	0.08	0.09	0.01	0.80
SUM	66.83	72.22	67.2	68.3	58.93	4.08	0.43

SFA (saturated fatty acids) 1:C8:0 + C10:0 + C12:0 + C13:0 + C14:0 + C15:0 + C16:0 + C17:0 + C18:0 + C20:0 + C21:0 + C22:0 + C23:0 + C24:0. MUFA (monounsaturated fatty acids) 2:C14:1 + C16:1 + C18:1n9c + C20:1 + C22:1n9 + C24:1. PUFA (Monounsaturated Fatty Acids) 3:C18:2n6c + C18:3n3 + C20:3n6 + C20:4n6 + C20:3n3 + C20:5n3 + C22:2 + C22:6n3. FISI: fresh soy oil + ITMs + I-Se; OISI: oxidized soy oil + ITMs + I-Se; FISY: fresh soy oil + ITMs + SY; OISY: oxidized soy oil + ITMs + SY; OOSY: oxidized soy oil + OTMs + SY.

**Table 6:** Effects of the quality of oil and source of trace minerals on fatty acid composition in the abdominal fat of growing–finishing pigs.

Items, mg/kg	FISI	OISI	FISY	OISY	OOSY	SEM	*p*-Value
SFA	441.83	389.31	414.18	416.41	413.37	8.67	0.47
C8:0	0.11	0.14	0.10	0.11	0.12	0.01	0.32
C10:0	0.88	0.98	0.90	0.93	0.93	0.02	0.55
C12:0	1.20	1.13	1.18	1.14	1.20	0.04	0.98
C13:0	0.01	0.01	0.02	0.02	0.01	0.01	0.97
C14:0	15.32	14.26	15.64	15.22	15.61	0.29	0.64
C15:0	0.56	0.57	0.59	0.66	0.67	0.61	0.94
C16:0	258.41	235.54	244.01	251.32	246.89	3.99	0.48
C17:0	3.82	3.67	3.30	4.10	4.21	0.27	0.91
C18:0	152.55	124.61	138.16	133.55	135.88	5.35	0.62
C20:0	2.47	1.88	2.79	2.26	1.67	0.16	0.25
C21:0	6.16	6.16	7.15	6.69	5.82	0.18	0.22
C22:0	0.16 ^ab^	0.13 ^c^	0.18 ^a^	0.17 ^ab^	0.14 ^ab^	0.01	<0.05
C23:0	0.13 ^ab^	0.17 ^ab^	0.10 ^c^	0.18 ^ab^	0.19 ^a^	0.11	<0.05
C24:0	0.06	0.05	0.10	0.05	0.04	0.01	0.33
MUFA	377.58	378.66	345.83	376.78	371.93	6.50	0.66
C14:1	0.15	0.18	0.17	0.17	0.20	0.01	0.69
C16:1	17.64	20.42	17.44	20.22	20.63	0.93	0.76
C18:1n9c	351.97	350.68	321.42	348.85	343.99	6.02	0.67
C20:1	7.52	6.91	6.43	7.19	6.51	0.25	0.69
C22:1n9	0.26	0.28	0.32	0.29	0.34	0.01	0.06
C24:1	0.04	0.19	0.06	0.05	0.27	0.05	0.61
PUFA	156.61	155.57	210.05	176.33	165.69	170.19	0.13
C18:2n6c	142.47	141.58	192.17	160.84	151.21	6.43	0.12
C18:3n3	8.71	8.23	11.59	8.96	8.23	0.43	0.14
C20:3n6	0.83	0.92	1.00	1.11	1.12	0.05	0.24
C20:4n6	2.25	2.57	3.08	2.67	2.92	0.14	0.44
C20:3n3	1.30	1.24	1.42	1.28	0.99	0.19	0.08
C20:5n3	0.20	0.17	0.18	0.24	0.24	0.02	0.63
C22:2	0.33	0.55	0.14	0.47	0.53	0.05	0.06
C22:6n3	0.53	0.30	0.51	0.76	0.47	0.07	0.44
SUM	976.51	924.08	970.83	970.03	951.58	9.51	0.42

SFA (saturated fatty acids) 1:C8:0 + C10:0 + C12:0 + C13:0 + C14:0 + C15:0 + C16:0 + C17:0 + C18:0 + C20:0 + C21:0 + C22:0 + C23:0 + C24:0. MUFA (monounsaturated fatty acids) 2:C14:1 + C16:1 + C18:1n9c + C20:1 + C22:1n9 + C24:1. PUFA (Monounsaturated Fatty Acids) 3:C18:2n6c + C18:3n3 + C20:3n6 + C20:4n6 + C20:3n3 + C20:5n3 + C22:2 + C22:6n3. ^a–c^ Different superscript within a row means significant different (*p* < 0.05); FISI: fresh soy oil + ITMs + I-Se; OISI: oxidized soy oil + ITMs + I-Se; FISY: fresh soy oil + ITMs + SY; OISY: oxidized soy oil + ITMs + SY; OOSY: oxidized soy oil + OTMs + SY.

## Data Availability

Data are contained within the article.

## Supplementary Materials

The following supporting information can be downloaded at: https://www.mdpi.com/article/10.3390/antiox13101227/s1, Table S1: The basal dietary composition and nutrients levels; Table S2: Effects of the quality of oil and source of trace minerals on short-chain fatty acids concentrations in colonic digesta in growing–finishing pigs.
