# Supplementary material for: Effects of Trace Mineral Source on Growth Performance, Antioxidant Activity, and Meat Quality of Pigs Fed an Oxidized Soy Oil Supplemented Diet
Source: Antioxidants (Basel). 2024 Oct 12;13(10):1227. doi: 10.3390/antiox13101227 (PMC11505604; doi:10.3390/antiox13101227)
Supplement: Supplementary file 1 [file antioxidants-13-01227-s001.zip › antioxidants-3223456-supplementary.pdf]

Table S1. The basal dietary composition and nutrients levels.

Ingredients	Dietary formulation，%
Corn	53.58
Wheat bran	25.00
Soybean meal	6.00
Corn DDGS	10.00
Soy oil (Fresh or oxidized)	2.00
Limestone	1.45
Lysine, 70%	0.70
NaCl	0.30
Dicalcium phosphate	0.25
Threonine	0.20
Tryptophan	0.02
0.5% Premix[1]	0.50
Total	100
Calculated nutrients levels, %	
metabolizable energy, kcal/kg	3542
Crude protein	14.22
SID Lys	0.94
SID Met	0.20
SID Trp	0.12
SID Thr	0.57

[1] The premix does not include Cu, Fe, Mn, Zn and Se. Premix provides: 50-75 kg per kg diet: Vitamin A, 5,600 IU; Vitamin D_3, 1,000 IU; Vitamin E, 21.6 IU; Vitamin K_3, 1.8 mg; Vitamin B_6, 1.8 mg; Vitamin B_{12}, 12 μg; Riboflavin, 4.0 mg; Thiamine, 0.88 mg; Niacin, 20 mg; Pantothenic acid, 10 mg; Folic acid, 0.4 mg; Biotin, 40 μg; Choline chloride, 320 mg. 75~100 kg and 100 kg~farming: Vitamin A, 5,200 IU; Vitamin D_3, 1,000 IU; Vitamin E, 17.2 IU; Vitamin K_3, 1.6 mg; Vitamin B_6, 1.6 mg; vitamin B_{12}, 10 μg; Riboflavin, 3.6 mg; Thiamin, 0.8 mg; Niacin, 17.6 mg; Pantothenic acid, 8.8 mg; Folic acid, 0.38 mg; Biotin, 32 μg; Choline chloride, 240 mg.

Table S2. Effects of the quality of oil and source of trace minerals on short-chain fatty acids concentrations in colonic digesta in growing-finishing pigs.

**Items, mg/kg**	**FISI**	**OISI**	**FISY**	**OISY**	**OOSY**	**SEM**	**P-value**
Lactate	57	55	73	58	61	11.5	0.83
Acetate	3626	3876	3497	3477	3253	240	0.48
Propionate	2100	2034	1926	2028	1863	156	0.83
Formate	55	48	54	58	67	10	0.71
Isobutyrate	224	227	226	229	230	17	0.99
Butyrate	1148	1166	1094	1204	1006	119	0.80
Isovalerate	210	175	189	161	185	26	0.75
Valerate	223	205	212	216	196	20	0.90
Total SCFA	7644	7789	7271	7434	6863	510	0.74

FISI: fresh soy oil + ITMs + I-Se; OISI: oxidized soy oil + ITM + I-Se; FISY: fresh soy oil + ITM + SY; OISY: oxidized soy oil + ITM + SY; OOSY: oxidized soy oil + OTM + SY.
